# Depressive and anxiety symptomatology among caregivers of children 0-3 years in Nairobi City County: Community-based prevalence study

**DOI:** 10.1371/journal.pgph.0006037

**Published:** 2026-04-20

**Authors:** Esther Jebor Chongwo, Japheth Adina, Kevinson Mwangi, Tabitha Shali, Edwin Dzoro, Cynthia Shitote, Vibian Angwenyi, Caroline Ngunu, Judy Macharia, Naomi Kigani, Rachel Odhiambo, Margaret Kabue, Amina Abubakar

**Affiliations:** 1 Institute for Human Development, Aga Khan University, Nairobi, Kenya; 2 Department of Clinical, Neuro- and Developmental Psychology, Faculty of Behavioural and Movement Sciences, Vrije Universiteit Amsterdam, Amsterdam, the Netherlands; 3 Department of Health, Nairobi City County Government, Nairobi, Kenya; 4 Centre for Geographic Medicine Research (Coast), Kenya Medical Research Institute/ Wellcome Trust Research Programme, Kilifi, Kenya; PLOS: Public Library of Science, UNITED STATES OF AMERICA

## Abstract

Caregivers of young children in low-resource urban settings face multiple stressors, which can affect their mental health. There is limited population-based evidence on the prevalence and correlates of depression and anxiety among caregivers of young children in these contexts. This study assessed the prevalence and associated factors of depressive and anxiety symptoms among caregivers of young children in Nairobi City County. We conducted a cross-sectional household survey with 2,903 primary caregivers of children 0–3 years. Depressive and anxiety symptoms were assessed using validated Swahili versions of the Patient Health Questionnaire-9 and the Generalised Anxiety Disorder-7, respectively, with a cut-off score of ≥10 indicating clinically relevant symptoms. Random intercept logistic regression models were fitted to assess the factors associated with depressive and anxiety symptoms while accounting for clustering within Nairobi City County’s sub-counties. Approximately 13.8% of caregivers had depressive symptoms, and 8.0% had anxiety symptoms. Stronger paternal involvement in childcare and parenting responsibilities was associated with lower odds of depressive and anxiety symptoms (adjusted odds ratio (aOR)=0.95 and aOR=0.93, both *P* < 0.001). Factors associated with higher odds of depressive and anxiety symptoms were pregnancy-related complications (depression: aOR=2.36, *P* < 0.001; anxiety: aOR=1.61, *P* = 0.003), and moderate household food insecurity relative to a food secure status (depression: aOR=3.67; anxiety: aOR=4.59; both *P* < 0.001). Higher wealth status was associated with lower odds of depressive and anxiety symptoms. A child’s history of hospital admission was additionally associated with higher odds of depressive symptoms (aOR=1.56, *P* = 0.018), while tertiary level education was associated with lower odds (aOR=0.65, P = 0.049). The noted prevalence of depressive and anxiety symptoms among caregivers in low-resource urban settings elucidates the need to integrate mental health services into the existing maternal and child health programmes in Kenya.

## Introduction

Mental health disorders are a major contributor to the global disease burden [1,2]. Depression and anxiety rank among the predominantly prevalent common mental health conditions, disproportionately affecting women [3]. A global systematic review estimated the prevalence of postnatal depression among women globally at 17.2% [4], with higher rates reported in low and middle-income countries (LMICs) [5], where socioeconomic factors, gender-based violence, and limited access to mental healthcare contribute to the elevated burden [6–8]. Notably, a recent review on the burden of postnatal depression in Sub-Saharan Africa (SSA) reported a pooled prevalence of 22.1%, with estimates ranging widely from 3.8% to 69.9% [9]. This is higher compared to estimates of 5–20% in high-income countries [10]. Other studies have similarly highlighted the high burden in the SSA region [6,11]. Similarly, global estimates suggest that approximately 18–25% of women experience anxiety during pregnancy, and around 15% affected during the postnatal period, with higher rates reported in LMICs [12]. The burden of mental health issues could be worse in urban settings due to a host of stressors, including economic hardship, inadequate housing, insecurity, barriers to quality and affordable health and social services, which may further increase the psychological burden on caregivers [13,14].

Existing evidence from Kenya reveals concerning trends. A study among 250 pregnant women in Nairobi’s informal settlements reported that 26.9% experienced antenatal depression and 6.4% had anxiety [15]. Similarly, a hospital-based study found that 16.2% of pregnant women had depressive symptoms and 6.6% exhibited anxiety symptoms [16]. The burden of depression and anxiety extends beyond pregnancy into the postnatal period. Depressive symptoms have been reported in 18.7% [17] and 27.1% [18] of mothers of young infants from low-resource settings attending maternal and child health clinics. Among post-natal mothers of severely malnourished children, the prevalence (64.4%) was concerning [19]. The variation in prevalence rates of depressive symptoms likely reflects differences in study design, sample size, screening tools, and different cut-off thresholds. Anxiety is also common during this period; in a recent study among mothers of pre-term infants in Kenya, 35.1% of caregivers screened positive for anxiety symptoms [20]. These high rates are driven by several risk factors, ranging from biological, psychological, and social factors [21]. Socioeconomic stressors such as poverty, food insecurity, and education [6,22–24], as well as psychosocial issues such as domestic violence and limited social support, are significantly linked to mental health disorders during the perinatal period [21,25,26]. Other factors have been reported, such as a history of mental illness, obstetric and perinatal complications, and poor child health [21,26–28]. Cultural beliefs and stigma surrounding mental illness present additional barriers to care-seeking and treatment, more so in low-resource settings [29]. In urban settings like Nairobi, added stressors may further heighten these risks.

Nairobi City County’s rapid urbanisation has placed immense pressure on housing, healthcare, and social infrastructure, exacerbating vulnerabilities among low-income populations [30,31]. Data from the 2022 Kenya Demographic Health Survey indicate that approximately 30% of women (15–49 years) had experienced physical violence [32]. The combination of urban stressors—including economic hardship, overcrowding, gender-based violence, and high rates of unemployment further increases the risk of mental health disorders among caregivers [33,34]. Given these challenges, Nairobi presents a critical setting for understanding the prevalence and correlates of depressive and anxiety symptoms, particularly in caregivers of young children in both formal and informal settlements. Caregiver mental health is important to study, as it has been shown to influence child outcomes [35–41]. Existing evidence on the burden of mental health in urban contexts, such as Nairobi, has mainly focused on the pre-natal populations, and is characterised by limited geographic coverage and reliance on facility-based assessments [17,18,42]. Beyond these, data on the impact of the COVID-19 pandemic on caregivers’ mental health remains scarce [43].

Although there is a high burden of mental health issues, services remain underdeveloped in SSA [44,45]. In Kenya, mental health remains under-prioritised, with limited integration into the essential package of reproductive, maternal, newborn, and child health services (RMNCH). A shortage of specialists, limited research, stigma, and inadequate screening further hinder early intervention [46,47]. This study adds to the literature by conducting the first population-based assessment of the mental health of caregivers with children aged 0–3 years across all sub-counties of Nairobi, encompassing diverse socio-economic strata and including both informal and formal urban settings. The findings will inform targeted interventions and policies to strengthen mental health services. This study aimed to assess the prevalence and correlates of depressive and anxiety symptoms in caregivers of young children (0–3 years) in Nairobi City County.

## Methods

### Study design

This study employed a cross-sectional household survey design. It was part of an initiative to evaluate the state of early childhood development (ECD) in Nairobi City County.

### Study setting

**Fig 1:**
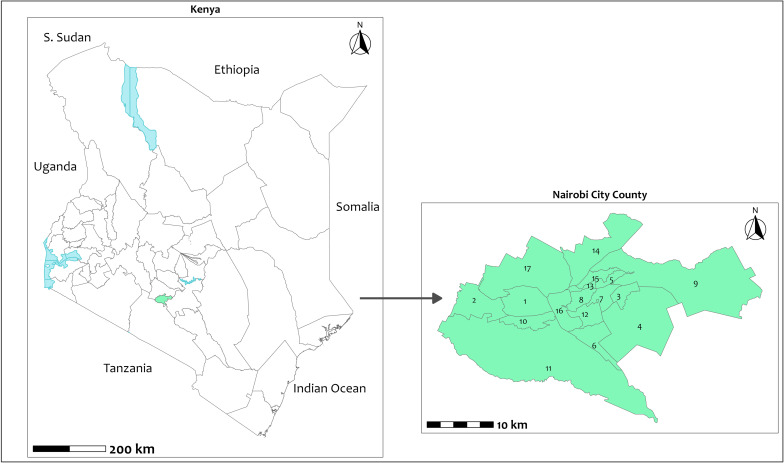
Map of Kenya showing the location of Nairobi City County. Map of Kenya showing the location of Nairobi City County. The map on the right shows the 17 sub-counties within Nairobi City County. The sub-counties are: 1 Dagoretti North, 2 Dagoretti South, 3 Embakasi Central, 4 Embakasi East, 5 Embakasi North, 6 Embakasi South, 7 Embakasi West, 8 Kamukunji, 9 Kasarani, 10 Kibra, 11 Langata, 12 Makadara, 13 Mathare, 14 Roysambu, 15 Ruaraka, 16 Starehe, and 17 Westlands. The map was author-created in R statistical software version 4.4.1. and shapefiles were obtained from the “geodata” package [51].

The study was conducted in Nairobi City County, Kenya’s capital and largest urban centre, which is home to an estimated 4.8 million people [48]. Approximately 60% (2 million) of Nairobi residents live in densely populated informal settlements [49]. The county is characterised by stark socioeconomic inequalities, with pockets of affluence alongside widespread poverty, food insecurity, and inadequate access to essential services [50]. Nairobi is administratively divided into 17 sub-counties and 85 wards, all of which were included in the survey sampling frame to ensure broad geographic and socioeconomic representation, as shown in Fig 1.

### Sample and sampling strategy

A total of 2903 primary caregivers with children (0–3) years old were recruited for the study. Primary caregivers included biological parents, or in their absence, adults who stay with the child on a full-time basis and were responsible for their daily care [52]. Notably, 95.8% of the participants were biological mothers of the children. Eligible participants were primary caregivers of young children between 0–3 years residing in Nairobi County and who could provide informed consent. Of those approached, 30 eligible participants refused to participate. Participants unavailable during the interview, unable to understand Swahili or English, or serving as temporary child caregivers were excluded.

Nairobi City County comprises 17 sub-counties, all of which were included, with sample sizes allocated proportionate to their estimated population sizes. Households were selected using a multi-stage cluster sampling approach, with sub-counties serving as the clusters. Within each sub-county, households with at least one child aged 0–3 years were randomly selected from routinely updated community health unit household listings maintained by the Nairobi City County community health strategy. In multi-household compounds, eligible households were enumerated, and one was randomly chosen. When multiple eligible caregivers or caregiver–child dyads were present within a household, one dyad was randomly selected to minimise intra-household clustering.

### Recruitment and data collection procedures

The initial step was study entry and engagement meetings with both county and sub-county officials to introduce the study and discuss the data collection approach. The community health services coordinators, including the community health services personnel (county and sub-county), community health assistants (CHAs), and community health promoters (CHPs), supported the identification and entry to households with eligible participants.

Forty-two trained field enumerators (25 males and 17 females) collected the data under the supervision of six field supervisors**.** The six supervisors (4 females and 2 males) had training in nursing (n = 2), public health (n = 1), statistics (n = 1), nutrition (n = 1), and sociology (n = 1). One supervisor was a diploma-trained registered nurse, while the remaining five held bachelor’s degrees. All had at least five years of research experience. The field enumerators held a degree or diploma-level qualification in social sciences, health, or a related field, with prior experience conducting survey research. They underwent five days of training covering survey objectives, survey tool administration, research ethics, including consent procedures, community entry, and navigating field challenges. They were trained on psychological first aid to deal with participants in need of mental health support. Emphasis was on survey tool administration and electronic data collection through the Open Data Kit (ODK) software in tablet offline mode. The training was led by the study investigators, including the principal investigator, and supported by team supervisors with extensive experience in maternal, child health and mental health research. To ensure clarity and appropriateness of the survey instruments, a one-day field pre-test was conducted among primary caregivers of young children in a neighbouring informal settlement not included in the main study site.

On the survey administration date, CHPs who work in and are part of the community accompanied field enumerators to households and introduced them to eligible participants. The field enumerators obtained written informed consent and collected the data in private spaces within the homes. Participants who screened positive for severe depressive or anxiety symptoms, expressed suicidal ideation, or disclosed safety concerns received immediate psychological first aid, after which the enumerator confirmed with the participant the need for referral. The participant’s information was then forwarded to the field supervisor, who coordinated with the CHAs to ensure referral to available mental health and psychosocial support services within Nairobi City County, including the nearest government-approved health facilities and community-based mental health resources. Data were collected between 25 September 2024 and 25 October 2024 electronically using ODK software (v2022.2.3) on password-protected tablets.

### Study measures

***Socio-demographic and health questionnaire:*** A structured, interviewer-administered questionnaire was used to collect data such as respondents’ age, occupation, marital status, number of household members, education level, and settlement type. The health history of the caregiver-child dyad was also asked. For example, the history of child admission, attendance of antenatal clinics, and whether the caregiver experienced complications during the pregnancy of the index child.

We used stunting and underweight to assess children’s nutritional status. The heights and weights of children were measured following the World Health Organisation (WHO) recommended procedures [53,54]. Weight was measured using mobile-calibrated SECA digital scales to the nearest 0.1 kg. Length/height was measured using a SECA length board to the nearest 0.1 cm, with recumbent length taken for children below two years and standing height for those above two years. The “zscorer” package in R statistical software was used to calculate height-for-age z-scores (HAZ) and weight-for-age z-scores (WAZ) [55]**.** We defined stunting and underweight as HAZ and WAZ scores 2 standard deviations below the median of the WHO 2006 Growth Standards Reference population [53].

An asset index scale was administered as a proxy for socio-economic status. We used variables employed by the Demographic Health Survey (DHS), including household asset ownership, house characteristics and quality, water and sanitation, in a principal component analysis framework [56] and categorised participants as having a wealth index status of either low, medium, or high.

***Caregivers’ mental health:*** The Patient Health Questionnaire (PHQ-9) [57] and Generalised Anxiety Disorder (GAD-7) scales [58] were administered to screen for depressive and anxiety symptomology, respectively. Swahili versions of these tools have undergone adaptation and validation among the adult population in Kenya [59,60]. A total score of 10 or higher (≥10) was set as the threshold for a positive screen for depressive and anxiety symptoms based on previous validations in East Africa [61]. The internal consistency in the current study was PHQ-9, a = 0.81; GAD-7, a = 0.85.

***Household Food Insecurity Access Scale (HFIAS):*** The HFIAS tool [62], consists of nine questions that explore food-related experiences over the previous four weeks, covering worries about food supply, inadequate food variety, and reduced food consumption and its physical effects [62]. Respondents indicated whether they experienced each situation and, if so, how frequently. Scores ranged from 0 to 27, with higher scores signifying more severe food insecurity. The HFIAS showed an excellent internal consistency in the study (a = 0.91). We further classified HFIAS into four categories: secure, mildly insecure, moderately insecure, and severely insecure [62] and used the categorised variable in the regression analyses.

***Paternal involvement:*** Paternal involvement was assessed using a 9-item scale designed to evaluate the extent of male caregiver support in childcare and parenting responsibilities. The tool was adapted from the UNICEF Multiple Indicator Cluster Survey (MICS) paternal involvement module [63] and customised for a study focusing on paternal engagement in Nairobi’s informal settlements, where it demonstrated acceptable internal consistency (Cronbach’s alpha = 0.77) [64]. This scale explored various domains of involvement, including engagement in playing and communication with the child, support for breastfeeding and infant feeding, contribution to household expenses and children#39;s education, accompaniment to perinatal clinics, and provision of psychological support. Male participants responded regarding their own involvement in caregiving and parenting responsibilities. The participants rated the frequency of their spouse#39;s/child#39;s father#39;s/male caregiver#39;s support for each item using a four-point Likert scale rating from never ‘0’ to always ‘3’. Responses were totaled to compute the paternal involvement scores, ranging from 0 to 27, with higher scores reflecting greater paternal involvement. The internal consistency in the current study was excellent (a = 0.89).

### Ethical considerations

Ethics approval was granted by the Institutional Ethics and Review Committee at Aga Khan University (ISERC) [Ref:2024/ISERC-136(V4)] and research authorisation from the National Commission for Science, Technology & Innovation (NACOSTI) [Ref: NACOSTI/P/24/40313]. Additionally, the participants gave written informed consent.

### Data analysis

Statistical analyses were conducted using R software version 4.4.1 [65]. Continuous variables were summarised using mean (standard deviation (SD), while frequencies (%) were used to describe categorical variables. The 95% confidence interval (CI) were computed to describe the prevalence of depressive and anxiety symptoms and comorbid depressive and anxiety symptoms in our sample, presented in summary tables.

Two separate multivariable mixed effects logistic regression models with random intercepts for sub-counties were used to assess the factors associated with a positive screen for depressive and anxiety symptoms. We used mixed-effects models to account for clustering within sub-counties. The models included child, caregiver, and household characteristics as the explanatory variables. Statistical significance was set at p-value <0.05. We report adjusted odds ratios (aOR).

It has been shown that it is appropriate to use PHQ-9 during the postnatal period [66]. However, our study sample included caregivers of children 0–3 years, and there was a chance for an inflated prevalence of a positive screen for depressive symptoms independent of affective symptoms due to the overlap between some PHQ-9 items with typical postnatal experiences, especially among mothers of infants. We therefore conducted a sensitivity analysis by stratifying prevalence by child age bands to assess whether the prevalence of a positive screen for depressive symptoms was elevated among mothers of infants relative to mothers of older children.

## Results

### Missing values

There were no missing values for either of the study’s outcome variables. However, some predictor variables had missing values as shown in Table 1. The proportion of missingness for these predictor values was < 5% and ranged between 0% to 2.5%. As a result, we conducted a complete case analysis.

**Table 1:** Participant sociodemographic characteristics.

Characteristic	N = 2,903
Child age (months), Mean (SD)	15.12 (9.8)
Child gender, n (%)	
*Female*	1,458 (50.2)
*Male*	1,445 (49.8)
Mother attended any ANC, n (%)	2,844 (98.0)
*Missing*	2
Place of delivery, n (%)	
*Home*	62 (2.1)
*Hospital/clinic*	2,834 (97.9)
*Missing*	7
Child ever admitted to a hospital, n (%)	270 (9.3)
*Missing*	1
Child birthweight, n (%)	
*Normal*	2,635 (91.8)
*Low*	236 (8.2)
*Missing*	32
Mother had problems during pregnancy, n (%)	718 (24.8)
*Missing*	9
Stunted, n (%)	623 (22.0)
*Missing*	73
Underweight, n (%)	284 (10.0)
*Missing*	60
Caregiver#39;s age (years), Mean (SD)	29.13 (7.0)
*Missing*	23
Relationship to the child, n (%)	
*Aunt/ Uncle*	10 (0.3)
*Biological father*	62 (2.1)
*Biological mother*	2,782 (95.8)
*Brother/ Sister*	2 (0.1)
*Grandparent*	46 (1.6)
*Step parent*	1 (0.0)
Marital status, n (%)	
*Single*	403 (13.9)
*Married/cohabiting*	2,230 (76.8)
*Separated/divorced/widowed*	270 (9.3)
Education level, n (%)	
*Primary or less*	820 (28.2)
*Secondary*	1,586 (54.6)
*Tertiary*	497 (17.1)
Employment status, n (%)	
*Not working*	1,597 (55.0)
*Working*	1,306 (45.0)
Wealth status, n (%)	
*Low*	707 (24.4)
*Middle*	1,481 (51.0)
*High*	715 (24.6)
Settlement type, n (%)	
*Formal*	1,142 (39.3)
*Informal*	1,761 (60.7)
No. of children ≤ 3 years, n (%)	
*1*	2,524 (88.0)
*2+*	343 (12.0)
*Missing*	36
Food security status, n (%)	
*Secure*	671 (23.1)
*Mild insecure*	464 (16.0)
*Moderately insecure*	1,668 (57.5)
*Severely insecure*	100 (3.4)
Household has a child living with disability, n (%)	54 (1.9)
*Missing*	11
Paternal involvement score, Mean (SD)	14.41 (8.2)

### Participants characteristics

Table 1 summarises sociodemographic characteristics of the 2903 child-caregiver dyads in the study. Most participants (95.8%) were the biological mothers of children, with a mean (SD) age of 29.1 (7.0) years, married or cohabiting (76.8%), and had attained secondary education (54.6%). Households were predominantly from informal settlements (60.7%), experienced moderate food insecurity (57.5%), and had a middle wealth index status (51.0%). The mean age of the children in the study was 15.1 months (SD = 9.8), with 50.2% being female. Most children (97.9%) were delivered in hospital or clinic settings.

### Prevalence of depressive and anxiety symptoms

**Fig 2:**
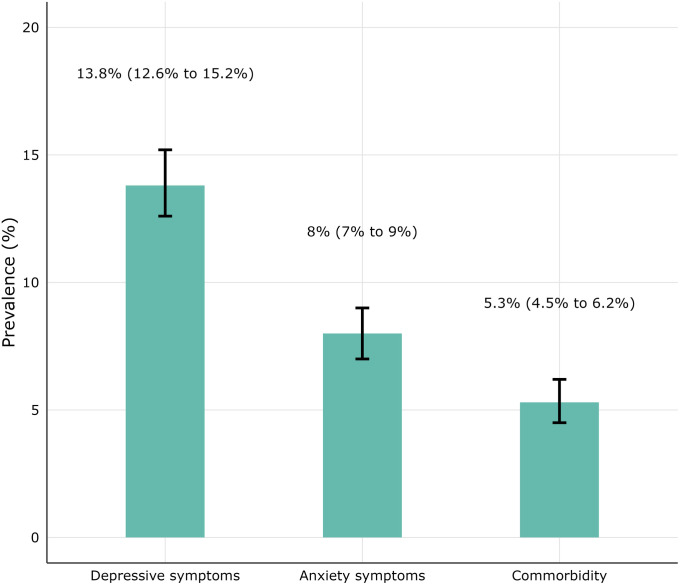
Prevalence of depressive and anxiety symptoms. Prevalence (95% CI) of depressive (PHQ-9 sum score ≥10), anxiety (GAD-7 sum score ≥10), and comorbid symptoms.

Fig 2 shows the proportion (95% CI) of participants in our sample with depressive symptoms, anxiety symptoms, and comorbid depressive and anxiety symptoms. Approximately 402 (13.8%; 95% CI: 12.6% to 15.2%) and 232 (8.0%; 95% CI: 7.0% to 9.0%) caregivers had depressive (PHQ-9 sum scores ≥10) and anxiety (GAD-7 sum scores ≥10) symptoms, respectively. Only 155 (5.3%; 95% CI: 4.5% to 6.2%) caregivers had comorbid depressive and anxiety symptoms. Approximately 281 (9.7%), 91 (3.1%), and 30 (1.0%) of the participants had mild, moderately severe, and severe depressive symptoms, respectively, while 169 participants (5.8%) had moderate anxiety symptoms, and 63 (2.2%) had severe anxiety symptoms (Table 2). Our sensitivity analysis suggested that the prevalence of a positive screen for depressive symptoms was relatively stable across child age bands with overlapping confidence intervals, with no indication of higher prevalence among mothers of infants (see S1 Fig).

**Table 2:** Severity of depressive and anxiety symptoms.

	n/N	Prevalence (%) (95% CI)
**Severity of depressive symptoms**		
None (0–4)	1524/2903	52.5 (50.7 to 54.3)
Mild (5–9)	977/2903	33.7 (31.9 to 35.4)
Moderate (10–14)	281/2903	9.7 (8.6 to 10.8)
Moderately severe (15–19)	91/2903	3.1 (2.5 to 3.8)
Severe (20–27)	30/2903	1 (0.7 to 1.5)
**Severity of anxiety symptoms**		
None (0–4)	1973/2903	68 (66.2 to 69.7)
Mild (5–9)	698/2903	24 (22.5 to 25.6)
Moderate (10–14)	169/2903	5.8 (5 to 6.7)
Severe (15–21)	63/2903	2.2 (1.7 to 2.8)

Notes: The numbers in brackets represent the range of sum scores for PHQ-9 and GAD-7, respectively.

### Factors associated with depressive and anxiety symptoms

#### Factors associated with depressive and anxiety symptom scores.

Results from the multivariable logistic regression models of the factors associated with a positive screen for depressive and anxiety symptoms are presented in Table 3. Caregivers of children ever admitted to a hospital had higher odds of depressive symptoms (aOR (adjusted odds ratio) =1.54; 95% CI:1.07 to 2.24, *P* = 0.018) relative to caregivers of children who had never been admitted. Tertiary education level was associated with lower odds of depressive symptoms (aOR =0.65; 95% CI: 0.42 to 0.99, *P* = 0.049) relative to primary education or less.

**Table 3:** Results from multivariable logistic regression models of factors associated with depressive and anxiety symptoms.

	Depressive symptoms		Anxiety symptoms	
Characteristic	adjusted OR (95% CI)	P-value	adjusted OR (95% CI)	P-value
Child age (months)	1.0 (0.98 to 1.01)	0.427	1.00 (0.98 to 1.01)	0.884
Child gender				
*Female*	—		—	
*Male*	1.01 (0.80 to 1.28)	0.926	0.88 (0.66 to 1.19)	0.415
Mother attended any ANC				
*No*	—		—	
*Yes*	0.94 (0.42 to 2.36)	0.889	1.64 (0.55 to 7.08)	0.436
Place of delivery				
*Home*	—		—	
*Hospital/clinic*	1.27 (0.58 to 3.14)	0.569	1.67 (0.57 to 7.17)	0.412
Child ever admitted to a hospital				
*No*	—		—	
*Yes*	1.56 (1.07 to 2.24)	**0.018**	1.55 (0.98 to 2.39)	0.055
Child birthweight				
*Normal*	—		—	
*Low*	0.77 (0.49 to 1.18)	0.246	0.68 (0.37 to 1.18)	0.191
Maternal complications during pregnancy				
*No*	—		—	
*Yes*	2.36 (1.84 to 3.03)	**<0.001**	1.61 (1.18 to 2.19)	**0.003**
Stunted				
*No*	—		—	
*Yes*	1.35 (1.00 to 1.81)	0.050	0.87 (0.58 to 1.28)	0.483
Underweight				
*No*	—		—	
*Yes*	1.11 (0.74 to 1.64)	0.617	1.42 (0.86 to 2.27)	0.157
Caregiver#39;s age (years)	1.01 (0.99 to 1.03)	0.280	0.98 (0.96 to 1.00)	0.097
Marital status				
*Married/cohabiting*	—		—	
*Single*	1.00 (0.65 to 1.52)	0.989	0.69 (0.40 to 1.16)	0.165
*Separated/divorced/widowed*	1.34 (0.87 to 2.04)	0.180	0.87 (0.51 to 1.47)	0.611
Education level				
*Primary or less*	—		—	
*Secondary*	0.88 (0.67 to 1.15)	0.337	0.85 (0.61 to 1.19)	0.339
*Tertiary*	0.65 (0.42 to 0.99)	**0.049**	0.83 (0.49 to 1.36)	0.466
Employment status				
*Not working*	—		—	
*Working*	1.09 (0.85 to 1.39)	0.507	1.07 (0.78 to 1.45)	0.679
Wealth status				
*Low*	—		—	
*Middle*	0.62 (0.47 to 0.81)	**<0.001**	0.68 (0.49 to 0.96)	**0.027**
*High*	0.67 (0.46 to 0.97)	**0.038**	0.63 (0.39 to 1.02)	0.064
Settlement type				
*Formal*	—		—	
*Informal*	1.00 (0.77 to 1.31)	0.993	0.74 (0.54 to 1.02)	0.062
No. of children ≤ 3 years				
*1*	—		—	
*2+*	1.21 (0.84 to 1.71)	0.290	1.07 (0.67 to 1.65)	0.773
Food security status				
*Secure*	—		—	
*Mild insecure*	1.62 (0.93 to 2.87)	0.092	0.70 (0.26 to 1.73)	0.447
*Moderately insecure*	3.67 (2.39 to 5.87)	**<0.001**	4.59 (2.64 to 8.70)	**<0.001**
*Severely insecure*	1.61 (0.64 to 3.67)	0.277	0.76 (0.12 to 2.87)	0.722
Household has a child living with disability				
*No*	—		—	
*Yes*	1.57 (0.75 to 3.13)	0.211	1.47 (0.57 to 3.32)	0.382
Paternal involvement	0.95 (0.93 to 0.97)	**<0.001**	0.93 (0.91 to 0.96)	**<0.001**

Abbreviations: CI = Confidence Interval, OR = Odds Ratio.

Common factors associated with both depressive and anxiety symptoms included a history of maternal complications during pregnancy, wealth status, food insecurity status, and paternal involvement. A history of maternal complications during pregnancy for the index child was associated with higher odds of depressive (aOR = 2.36; 95% CI: 1.84 to 3.03, *P* < 0.001) and anxiety (aOR=1,61; 95% CI: 1.18 to 21.9, *P* = 0.003). Caregivers from households with a high (aOR = 0.67; 95% CI: 0.46 to 0.97, *P* = 0.038) and middle wealth status (aOR = 0.67; 95% CI: 0.46 to 0.97, *P* = 0.038) had lower odds of depressive symptoms while those from households with middle wealth status also had lower odds of anxiety symptoms (aOR=0.68; 95% CI: 0.49 to 0.96, *P* = 0.027) compared to those from low-wealth households. In addition, compared to those with a secure food insecurity status, the odds of depressive symptoms and anxiety symptoms were 3.67 (95% CI: 2.39 to 5.87, *P <* 0.001) and 4.59 (95% CI: 2.64 to 8.70, *P <* 0.001) times higher among those from moderately food insecure households, respectively. Finally, paternal involvement was associate with lower odds of depressive and anxiety symptoms with a unit increase in paternal involvement scores associated with 5.0% (95% CI: 3.0% to 7%), *P* < 0.001) and 7.0% (95% CI: 4.0% to 9%, *P* < 0.001) lower odds of depressive and anxiety symptoms, respectively (Table 3).

## Discussion

This study examined the prevalence and correlates of depressive and anxiety symptoms among caregivers of children 0–3 years in Nairobi, Kenya. Notably, 13.8% of caregivers screened positive for depressive symptoms and 8.0% for anxiety symptoms. The majority of those with depressive symptoms experienced mild to moderate severe symptoms, whereas most of those with anxiety symptoms reported mild to moderate levels of severity. Several factors were associated with caregiver mental health outcomes. For instance, pregnancy-related complications, lower household wealth status, household food insecurity, and low paternal involvement emerged as common correlates of both depressive and anxiety symptoms. Additional predictors specific to depressive symptoms included a history of child hospitalisation and a lower education level.

The observed rates of depressive and anxiety symptoms in our study align with global estimates from other LMICs, where the prevalence of perinatal mental health issues is disproportionately higher [5]. The prevalence of anxiety symptoms observed in our study (8.0%) is comparable to previous estimates of 6.4% from a community-based cross-sectional analysis of pregnant women in their third trimester residing in an urban informal settlement in Nairobi City County [15] and 6.6%, from a cross-sectional study among urban-based pregnant women in their second and third trimesters attending antenatal care clinic [16]. This suggests a consistent pattern of anxiety symptoms during the perinatal period in Kenya. Interestingly, the prevalence of depressive (13.8%) and anxiety (8.0%) symptoms found in our study is lower than some previous estimates from Nairobi, particularly among specific high-risk groups. For instance, earlier studies reported higher rates of depression in pregnant women (26.9%) [15], and postnatal mothers (18.7%–64.4%) in urban informal settlements and public hospitals [17–19]. Although our estimates are somewhat lower, they still indicate a notable mental health burden among caregivers of young children in Nairobi. The observed difference could reflect our population-based sampling across both formal and informal urban settings, offering more generalizable estimates of the burden of depressive and anxiety symptoms within the general caregiving population in Nairobi than previous studies limited to clinical or high-risk groups.

Caregivers of children who experienced complications during pregnancy reported significantly higher depressive and anxiety symptom scores, suggesting that perinatal maternal complications can have lasting effects on a caregiver’s psychological well-being, potentially heightening vulnerability to both depressive and anxiety symptoms after childbirth [67]. History of maternal complications during pregnancy, such as pre-existing health conditions or pregnancy-related complications, may contribute to both physical and emotional strain, influencing mental health outcomes [68]. In the present study, pregnancy-related complications were assessed using caregiver self-report of problems experienced during the index pregnancy and not specific clinical diagnoses. Similar associations between pregnancy-related health complications and poorer maternal mental health outcomes have been reported in Kenya [15]. These results underscore the need of addressing maternal health complications not only to improve physical outcomes, but also to protect the long-term mental health of caregivers and child outcomes. Future research would benefit from more detailed studies, including those from longitudinal designs, to elucidate the temporal pathways linking adverse pregnancy experiences to caregiver mental health.

Marital status was not significantly associated with either depressive or anxiety symptoms in the multivariable analysis. Although caregivers who were separated, widowed, or divorced had higher odds of depressive symptoms compared to those who were married or cohabiting, these associations were not statistically significant, and no differences were observed for anxiety symptoms. These findings contrast with previous studies reporting a protective effect of marriage [69]. In urban informal settlements, high levels of gender-based violence, relationship conflict, and economic stress may undermine the potential mental health benefits of marriage [70]. Exposure to intimate partner violence has been consistently associated with increased risk of depression and anxiety among women, including during the perinatal and caregiving periods. As such, being married or cohabiting may not confer psychological protection where relationships are characterised by instability or violence [25,71]. However, this study did not collect data on gender-based violence or relationship quality, limiting our ability to directly assess whether these factors modified the association between marital status and caregiver mental health. Our findings suggest that marital status alone may be an inadequate proxy for psychosocial support, highlighting the need for future research to incorporate measures of intimate partner violence, relationship quality, and social support networks when examining caregiver mental health, particularly in urban settlements.

Notably, we found that stronger male involvement in childcare and parenting responsibilities was associated with lower odds of depressive and anxiety symptoms. These results concur with previous studies from LMICs reporting that male involvement is linked to improved mental health outcomes [72,73]. Increased male involvement in caregiving responsibilities can support maternal mental health by strengthening coparenting relationships, improving the quality of the couple’s partnership, and redistributing childcare responsibilities, which may help reduce maternal stress by creating opportunities for women to rest, pursue personal interests, or engage in paid work, all of which can promote better psychosocial well-being [74–77]. Overall, these findings underscore that fostering greater male engagement in ECD may be a key strategy to support family well-being.

In many low- and middle-income settings, including SSA, food insecurity, limited access to health care, poverty, and high caregiving burden frequently co-occur, contributing to psychological distress among caregivers [1,21,23,78,79]. We found that depressive and anxiety symptoms were significantly more common among caregivers experiencing moderate food insecurity compared with those from food-secure households. These findings align with prior research demonstrating that food insecurity and limited socioeconomic resources are associated with poorer mental health outcomes [80–83]. This may be explained by the fact that caregivers face psychological stress, including feelings of shame and inadequacy, when they are not able to provide the basic needs of their families, which in turn leads to symptoms of depression and anxiety [84]. Similarly, caregivers from higher-wealth households had lower odds of both depressive and anxiety symptoms, highlighting the protective role of economic resources in buffering against psychological distress. These results underscore the need for addressing socioeconomic vulnerabilities, including poverty and food insecurity, to support caregiver mental health [85].

Other factors that were significantly associated with depressive symptoms included recent child hospitalisation and caregiver education level. Caregivers whose children had been recently hospitalised reported higher depressive symptom scores, which is consistent with prior research [86,87], and may reflect the psychological and financial strain that they encounter when their children are unwell. Further, caregivers with tertiary-level education compared to those with primary or less had lower odds of depressive symptoms, consistent with previous research [88]. This suggests the potential protective role of education, possibly through improved health literacy, problem-solving skills, access to resources, and coping capacity.

### Implications for policy and practice

The findings of this study have important implications for policy and practice in urban low‑resource settings such as Nairobi. First, the burden of depressive and anxiety symptoms among caregivers underscores the urgent need to integrate routine mental health screening, counselling, and referral services into existing maternal, newborn, and child health and ECD programmes, particularly at the primary health care and community levels. Given the strong associations observed with food insecurity and household wealth, mental health interventions should be embedded within broader social protection and poverty‑alleviation initiatives, including food assistance and cash‑transfer programmes, to address the underlying social determinants of caregiver mental health. In addition, the protective role of paternal involvement highlights the importance of policies and programmes that actively promote male engagement in caregiving and parenting, such as father‑inclusive antenatal and postnatal services, parenting programmes, and gender‑transformative interventions. Strengthening support for caregivers who experience pregnancy‑related complications or child hospitalisation, through enhanced follow‑up, psychosocial support, and linkages to mental health services, may further mitigate psychological distress. Collectively, these findings support a multisectoral approach that integrates mental health, social protection, and family‑centred interventions to improve caregiver well‑being and, ultimately, child development outcomes in urban settings.

## Strengths and limitations

This is the first and the largest population-based survey of depressive and anxiety symptoms among primary caregivers with young children (0–3 years) in Nairobi, covering both formal and informal settlements. We had a large sample size which strengthens the representativeness and generalizability of the findings. The study also used adapted and validated tools to assess depression and anxiety ensuring greater reliability of the results. Furthermore, the inclusion of diverse sociodemographic and contextual variables, such as paternal involvement, maternal health, and food insecurity, provides further insights into the factors associated with caregiver mental health.

However, the study also has its limitations. Its cross-sectional design prevents any causal inferences and limits the ability to determine the directionality of associations between the identified factors and caregiver mental health outcomes. Also, the utilisation of self-reported data may have introduced social desirability bias. Additionally, the lack of a follow-up clinical assessment limits the interpretation of the extent of depression and anxiety among caregivers, as symptom-based screening may not fully capture clinically significant cases. These results should therefore be interpreted with caution. Nonetheless, the study provides valuable insights for informing targeted interventions to support caregiver mental health in urban low-resource settings.

## Conclusion and recommendations

Caregivers of young children in Nairobi experience a notably high burden of depressive and anxiety symptoms, with key factors such as maternal complications during pregnancy, low paternal involvement, marital disruption, and food insecurity emerging as significant predictors. Additionally, child hospitalisation, caregiver employment, and having a child with disability were associated with higher anxiety symptoms. These findings emphasise the need to prioritise caregiver mental health as part of ECD strategies. We recommend integrating mental health programmes into existing maternal and child health services by establishing routine screening, counselling, information-education sessions and referral systems. Doing so can help raise awareness, reduce stigma, and encourage caregivers to seek support. Strengthening caregiver well-being will also require scaling up parental counselling and coaching interventions delivered through health facilities, community groups, home visits, and digital platforms. Moreover, promoting male involvement and ensuring tailored support for caregivers facing complex challenges can enhance family resilience and child development outcomes. Finally, further research is needed to assess the long-term effects of caregiver mental health on child development and evaluate the impact of scalable, context-specific mental health interventions in urban low-resource settings.

## Supporting information

S1 Fig Prevalence of a positive screen for depressive symptoms among caregivers by child age band. (DOCX)

S2 Fig Participant flow diagram. (DOCX)

S1 Table STROBE Checklist. (DOCX)
