# Supplementary material for: Depressive and anxiety symptomatology among caregivers of children 0-3 years in Nairobi City County: Community-based prevalence study
Source: PLOS Glob Public Health. 2026 Apr 20;6(4):e0006037. doi: 10.1371/journal.pgph.0006037 (PMC13094971; doi:10.1371/journal.pgph.0006037)
Supplement: S1 Fig — (DOCX) [file pgph.0006037.s001.docx]

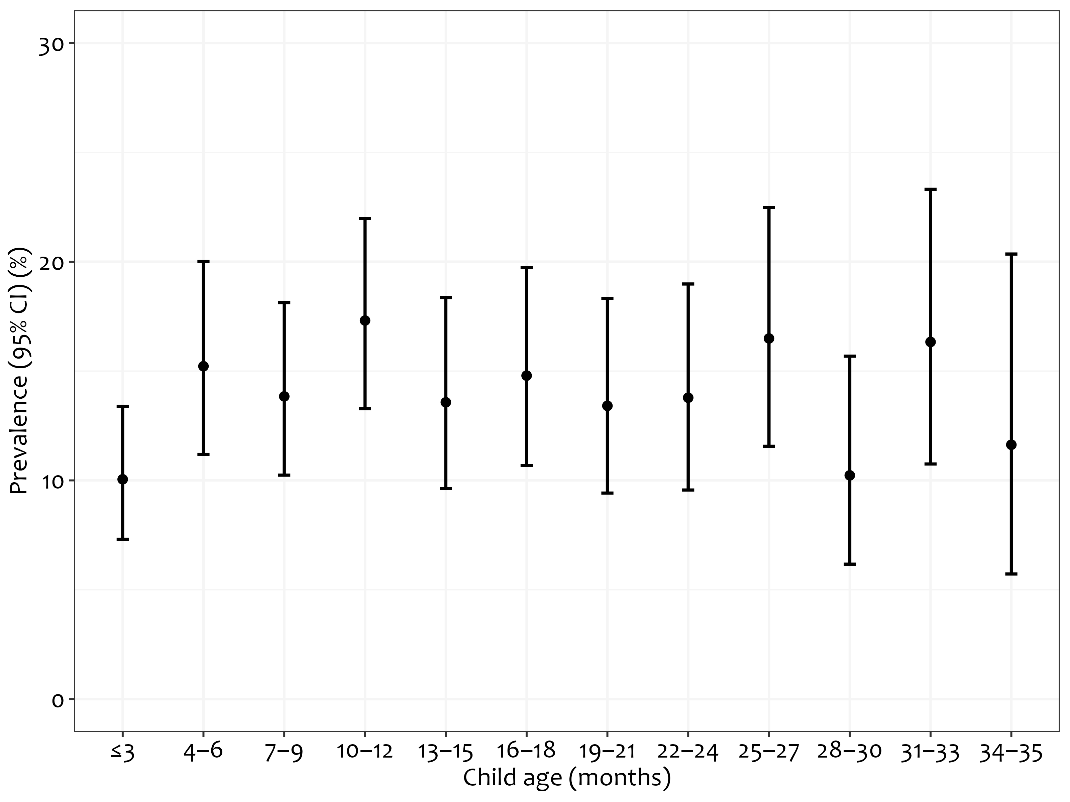


**S1 Fig**: Prevalence (95% CI) of a positive screen for depressive symptoms among caregivers by child age band. The 95% CIs were derived from the binomial exact test.
