# Supplementary material for: Depressive and anxiety symptomatology among caregivers of children 0-3 years in Nairobi City County: Community-based prevalence study
Source: PLOS Glob Public Health. 2026 Apr 20;6(4):e0006037. doi: 10.1371/journal.pgph.0006037 (PMC13094971; doi:10.1371/journal.pgph.0006037)
Supplement: S2 Fig — (DOCX) [file pgph.0006037.s002.docx]

**S2 Fig***:* **Participant flow diagram**

Households approached
(n = 2933)

Consented and assessed
(n = 2903)

Included in analysis
(n = 2903)

Refusals
(n = 30)
